# The Influence of Dentin Age and the Presence of Cracks in Removal of the Root Filling Material

**DOI:** 10.22037/iej.v13i3.20291

**Published:** 2018

**Authors:** Lilian Rachel de Lima Aboud, Ricardo Tadeu Lopes, Bernardo Camargo dos Santos, Thaís Maria Pires dos Santos, Leonardo Aboud Costa Viana, Miriam F Zaccaro Scelza

**Affiliations:** a *Department of Endodontics, Fluminense Federal University (UFF), Brazil; *; b *Department of Nuclear Energy, Federal University of Rio de Janeiro (UFRJ), Brazil*

**Keywords:** Dentin, Microcomputed Tomography, Retreatment

## Abstract

**Introduction:**

This study evaluated the removal of the filling material during endodontic retreatment considering the presence of cracks and the dentin age.

**Methods and Materials:**

A total of 20 freshly extracted single-rooted teeth were categorized into the following two groups according to the age of the patients: Group Young (Y; aged 18-30 years) and Group Old (O; aged ≥60 years). Each tooth specimen was scanned by microcomputed tomography (micro-CT) subsequently after endodontic retreatment with the Reciproc instruments (REC). The images were analyzed for differences in the volume of dentin cracks and the presence of the filling material in the middle and apical thirds of the teeth among the groups, according to the dentin age.

**Results:**

The micro-CT images showed that after retreatment, there were more cracks in the old root dentin than those in the young root dentin, although the difference was not statistically significant (*P*>0.05). The greatest reduction in the filling material was achieved when the old root dentin with cracks was retreated when compared with that of the young root dentin with cracks, but the difference was not statistically significant (*P*>0.05).

**Conclusion:**

The dentinal age and the presence of cracks were not found to be relevant factors for the removal of the filling material.

## Introduction

Non-surgical endodontic retreatment can be indicated in case of treatment failures [1, 2]. Efficiency in the removal of the filling material from the interior part of the root canal and the preservation of the dentin structure are fundamental for successful retreatment [3]. However, it is known that dentin undergoes modifications with age, especially dehydration. The old dentin is less hydrated than the young dentin, which contributes to the development of cracks on the dentinal surface that can lead to endodontic failure [4, 5].

Several techniques involving the use of solvents, heat, mechanical instruments, and combinations of different methods can be used for the removal of the filling material [6]. Reciprocating instruments have been widely used for this purpose as they appear to cause less damage to the dentin structure [7, 8]. The use of reciprocating instruments has been considered as the most rapid method for removing gutta-percha and sealer when compared with rotating files [9-11] besides extruding less apical debris [12]. The Reciproc system uses a single file and has been selected because of its flexibility due to the fact that the system is made up of M-Wire nickel-titanium alloy subjected to thermal treatment with an S-shaped cross-section, which can reduce the incidence of cracks when compared with the rotary system [13, 14]. However, the behavior of this type of file has not yet been analyzed, taking into account the dentinal age factor.

The dentin structure and the removal of the filling material from the interior parts of the root canal have been analyzed through image analysis using stereomicroscopy, optical microscopy, electronic microscopy, conventional tomography and x-rays. However, microcomputed tomography (micro-CT) imaging has been widely used today as it provides accurate details of the defects with high-resolution images, especially in endodontic studies [15-18].

**Figure 1:**
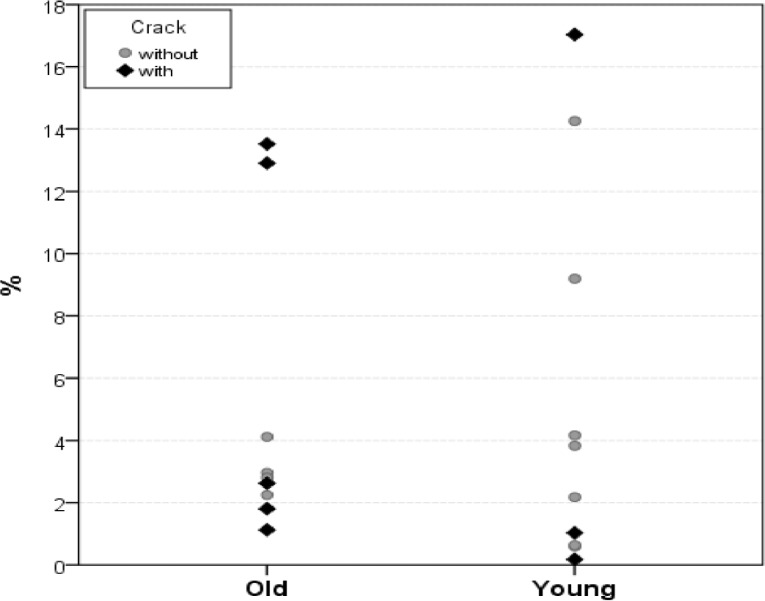
Demonstrative graphic showing the percentile of the remaining filling material (%) in the Old and Young dentin according to the presence of cracks

This study was conducted to evaluate the removal of the filling material using the Reciproc R25 (25/0.08) file by micro-CT imaging, taking into account the age of the dentin and the presence of cracks.

## Materials and Methods

***Selection of samples and micro-CT specifications***

After obtaining the approval of the Ethics Committee of the Federal Fluminense University, Niterói, Brazil (local ethical committee review number 40186714.5.0000.5243) the number of samples was selected according to a statistical database. A total of 20 freshly extracted mandibular single-rooted human teeth, with single canal, extracted for reasons not related to this study, were selected for this *in vitro* study. According to the Bank of Teeth at Federal University of Juiz de Fora, MG- Brazil, the specimens were cleaned, washed, dried and immersed in 2% chlorhexidine solution (Consepsis V, Ultradent Products, Inc., South Jordan, UT, USA). After that, the teeth were stored in artificial saliva at 37^°^C throughout the experimental procedures. Teeth with severe radicular curvature, root fractures, curved canals, caries or restorations and severe calcified and multiple roots were excluded. The specimens were categorized into the following two groups: Group Young (Y), which included teeth belonging to patients aged 18-30 years (*n*=10), and Group Old (O), which included teeth obtained from patients aged ≥60 years (*n*=10). A micro-CT scanner (Skyscan 1173; Brucker micro-CT, Kontich, Belgium) operating at 70 kV and 114 mA was used for evaluating the dentinal cracks and the removal of the filling material. A specific metal support was used and an acrylic resin base was prepared so that each dental specimen remains securely fitted for further observations. A total of 449 micro-CT slices (thickness, 14. 25 µm each) were analyzed from samples of the apical and middle thirds of the teeth. Thus, a profile of approximately 15 mm from the apex of the root was obtained. 

**Figure 2:**
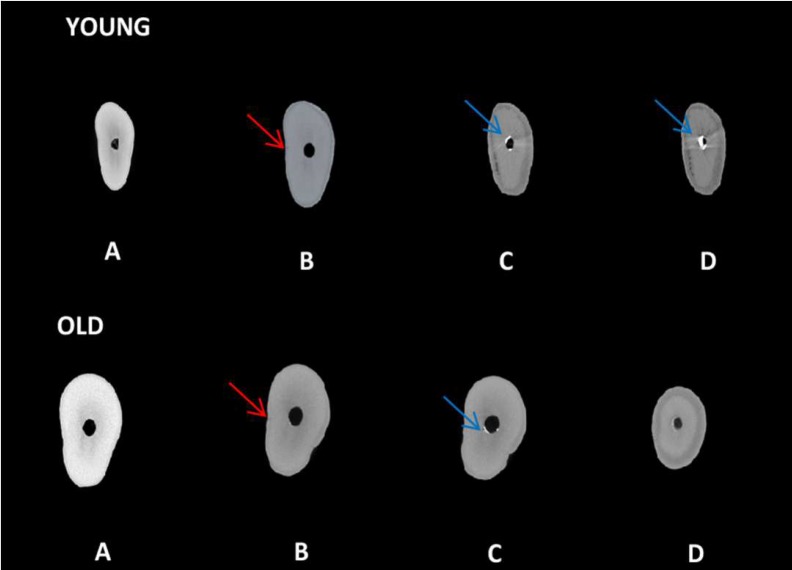
Micro-CT image 2D of Young and Old dentin*: A)* Initial; *B)* After retreatment medium third with crack (red arrow); *C) *After retreatment medium third with remaining material filling (blue arrow); *D)* After retreatment of apical third with or without remaining material filling (blue arrow

***Root canal preparation***

Before starting the endodontic treatment, to simulate the periodontal ligament space, the surface of the roots was coated with a polyether-based impression material (Impregum F, 3MESPE, St Paul MN, Germany). Then, it was embedded in a base with a diameter of 18 mm made up of epoxy resin [18].

Root canal instrumentation procedures were performed using the reciprocating system namely R25 (25/0.08) file (VDW, Munich, Germany) using the motor VDW (Silver; VDW GmbH, Munich, Germany) according to the manufacturers’ instructions. To ensure standardization, the teeth were accessed and apical patency was performed with a #15 K-file (Dentsply Maillefer, Ballaigues, Switzerland) until its file was visible at the apical foramen, so the working length was stipulated 0.5 mm shorter than this measurement. To prepare each canal, one Reciproc R25 file was used and it was used only once in each canal during 30 sec, being carefully inserted up to the work length. An abundant irrigation aspiration with 5.25% sodium hypochlorite (NaOCl; Formula e Ação Farmácia, São Paulo, Brazil) was performed throughout the instrumentation process. After the instrumentation, copious final irrigation was performed by inserting a syringe and endodontic needle (Navitip ULtradent, São Paulo, Brazil) into the root canal to the working length. A total of 30 mL of 5.25% NaOCl, followed by a final rinse with 10 mL of 10% citric acid (Formula e Ação Farmácia) and 5 mL of double-distilled water. Then, the samples were randomly assigned to one to two groups, categorized according to age, for the next step.

**Figure 3:**
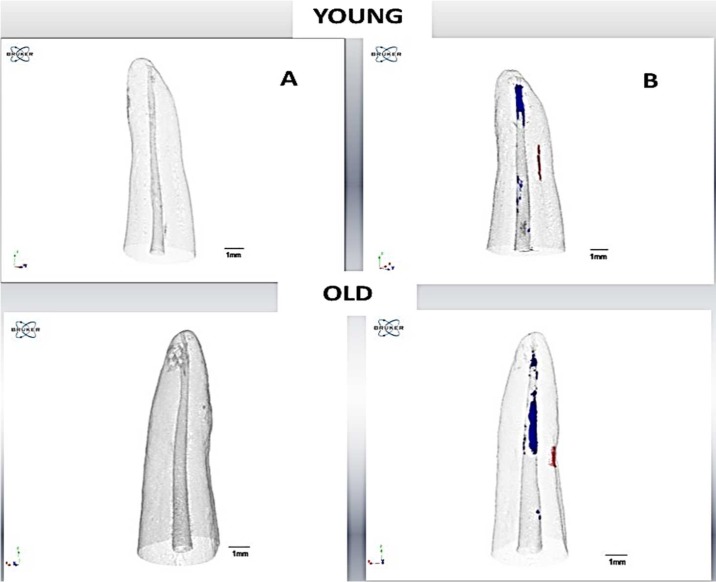
Micro-CT image 3D Young and Old dentin: *A)* Initial; *B)* After retreatment: cracks in red and remaining material filling in blue

***Root canal filling***

The specimens were dried using Reciproc R25 paper cones (VDW, Munich, Germany). The canals were filled with Reciproc R25 gutta-percha (VDW, Munich, Germany) and AH-Plus sealer that was handled according to the manufacture’s specifications (Dentsply De Trey, Konstanz, Germany). With the intention of causing less impact on the root and reducing the risk of crack formation at this stage, the warm vertical compaction technique using a single-cone R25 (Easy Thermo- Pack, Easy Endo, Belo Horizonte, Brazil) was used [19]. The quality and apical extent of the root canal filling were assessed through periapical x-rays. A temporary restoration (Coltosol, Vigodent, Rio de Janeiro, RJ, Brazil) was used for the coronal sealing. After this process, the teeth were immersed in artificial saliva at 37^°^C for 30 days.

***Removal of the filling material***

The Y and O groups were retreated. The bulk of the gutta-percha in the coronal third of the canal was removed using an electric heat carrier (Easy Thermo-Pack, Easy Endo, Belo Horizonte, Brazil). The REC R25 file was introduced into the canal in a slow in-and-out pecking motion by applying slight pressure, without pulling the instrument completely out of the canal or advancing into the filling material toward the apical third during 30 sec within the root canal. The Reciproc R25 file was used only once in each canal and only one root canal was retreated with a file.

Hence, a total solution volume of 45 mL was used per root canal (30 mL of 5.25% NaOCl, 10 mL of 10% citric acid and 5 mL double-distilled water). The coronal sealing was carried out using Coltosol. Subsequently, the teeth were subjected to micro-CT analysis to detect the removal of the filling material taking into consideration the presence of cracks and the age of the dentin.

A total of 449 frames (thickness 14.25 µm) obtained from the medium and apical thirds of the root of each tooth were evaluated. The software CTAn Skyscan Bruker (Bruker micro-CT, Kontich, Belgium) was used for the processing of images. Frame integration functions of the Image Pro Plus 7.0 software (Media Cybernetics, Bethesda, USA) were used for assessing crack formation and visualization of remaining root canal filling. This software enables characterization and counting of objects over fifty manual and automatic measurement tools including areas, perimeters, lengths, roundness, major and minor axes and angles. Besides that, the animated visualization of images along the axis of the tooth, from the start of crack formation to the end was possible through this software. The frames were analyzed by three evaluators until reaching an agreement.

***Statistical analysis***

Data obtained from the volumetric analysis of the residual filling material were statistically compared using non-parametric tests. The software used was SPSS (SPSS version 17.0, Chicago, IL, USA). The Mann-Whitney test was used with a significance level of 0.05 for comparisons of the mean presence of cracks and the removal of the filling material between the young and the old root dentin that were retreated with Reciproc. 

## Results

The incidence values of the presence of cracks and the percentile values of the remaining filling material are shown in Table 1. In the retreatment analysis (by micro-CT), Group O showed more volume of cracks than that of Group Y, with no statistical significance. In relation to the removal of the canal filling, Group Y presented more percentile of the remaining filling material than that of Group O also with no statistical significance (*P*>0.05) (Figure 1). 

## Discussion

Removal of the root canal filling material and the proper cleaning of dentinal walls are the principal objectives of endodontic retreatment [2, 20]. Due to the dental anatomy, the apical portion has been the most difficult part to remove the filling material, which can lead to the formation of dentin cracks [21]. The results of the present study corroborate with those reported by Taha *et al*. [21] based on the presence of a remaining of the filling material in the apical third. Figures 2 and 3 show the remaining of the filling material in the apical third after the retreatment procedure. The micro-CT technology allows for a detailed analysis of the roots before performing any endodontic procedure [22, 23]. 

Some factors may alter the *in vitro* dentin structure leaving the search results vulnerable. For example, the storage conditions are known to affect the incidence of dentinal cracks before and during endodontic procedures [24, 25]. In this study, the dental specimens were stored in artificial saliva at 37^°^C throughout the study period because they are needed to be well hydrated for simulating clinical circumstances. The use of irrigating substances, such as NaOCl, during the endodontic process can also induce defects in the dentinal surface [26]. In this study, 10% citric acid was chosen because EDTA (17%) has been shown to induce significant demineralization in the old root dentin during the final irrigation [27]. The simulating periodontal ligament is another means of minimizing the effect of defect-causing factors to neutralize the impact on the dentin during the endodontic procedures [13]. In addition, some studies have reported about the effect of rotary and reciprocating system files on the incidence of dentinal defects during endodontic procedures [7, 22, 28]. 

**Table 1:** Percentile values of the remaining filling material and the presence of cracks after endodontic retreatment in the Old and Young groups

**Values of remaining filling material and presence of cracks ** **(%)**			
**Teeth**	**Remaining filling material (%)**		
	**Old**	**Young**	
1	2.97	17.04*	
2	2.25	0.18*	
3	1.81*	9.19	
4	1.13*	0.60	
5	2.63*	3.83	
6	4.12	1.03*	
7	12.90*	14.26	
8	13.52*	2.18	
9	2.81	4.17	
10	0.00	0.64	
**Mean**	4.90	5.31	
**Mean (SD)**	2.81 (4.78)	3.01(6.10)	
***P*** **-value Mann-Whitney test**	0.683		
**Cracks ** **(%)** **	55.6%		30.0%

* *Teeth with cracks;*

** *P-value exact for Fisher Test to comparison between the cracks proportions = 0.370*

Several studies have shown that the choice of the instrument used in the removal of the filling material has an influence on the formation of dentin defects [7, 8, 13, 18, 19, 22-26]. The choice of the instrument was based on studies that showed that the reciprocating system caused less damage to the dentin structure and efficiently removed the filling material. The REC file is safe for the removal of fillings in straight root canals and requires short periods of time during the retreatment procedure, and the file can be kept more centered in the canal [9, 29-32]. The REC R25 file was used according to the anatomy of the chosen specimens.

To increase our knowledge, this *in vitro* study evaluated the remaining filling material taking into account the presence of dentinal cracks and whether there was any correlation between the young and the old root dentin. Although the literature describes that young hydrated dentin presents certain mechanisms that contribute to the dissipation of energy and resistance to the development of cracks on the surface and that the crack resistance of human dentin decreases with tissue age and dehydration [4, 5], the present study has indicated through the retreatment analysis by micro-CT that Group O showed more presence of cracks than Group Y, but with no significant difference (*P*>0.05).

The removal of the filling material from the root canal can be influenced by the anatomy of the chosen type of teeth. The studies of Rosa *et al*. [17] and Taha *et al*. [21] evaluated the removal of the filling material due to curvature complexity. In the present study, single-rooted, lower incisor teeth were chosen as they have straight roots, which reduce the curvature variables, which could cause complications in the retreatment process. It is worth mentioning that the influence of the dentin age was a differential parameter in the proposed methodology. A greater percentage of the removal of the filling material was observed in the Group O that had cracks, although not statistically significant (*P*>0.05). The presence of root canal ramifications and apical deltas in the apical region and the removal of the smear layer can cause the sealer to penetrate into these ramifications, which causes the residual filling of the canal material after re-treatment and does not have any relation to the cracks. Besides that, the presence of an isthmus in the mandibular anterior teeth or in untouched areas can simulate the crack view which can be a limitation of our study [33]. In order to minimize errors related to the presence or absence of cracks, three examiners did the analysis of the images separately. The results were compatible between the presence and absence of cracks. The results of the present study suggest that dentin age along with the presence of cracks does not interfere with the removal of the filling material.

## Conclusion

Within the limitations of the present study, the dentinal age and the presence of cracks were not found to be relevant factors for the removal of the filling material.
